# Novel Partial Loss-of-function *STAT3*-variant as Cause of Hyper-IgE-Syndrome in a Danish Family with Variable Expressivity

**DOI:** 10.1007/s10875-026-02036-8

**Published:** 2026-06-08

**Authors:** Camilla Heldbjerg Drabe, Jonathan Gehrig, Jens Magnus Bernth Jensen, Mira Marie Laustsen, Carsten Schade Larsen, Camilla Darum Sørensen, Kirstine Overgaard Dyrmose, Sophia Hammer-Hansen, Jens Wittner, Tania Masmas, Hanne Marquart, Stephan Ehl, Line Borgwardt, Terese L. Katzenstein

**Affiliations:** 1 Department of Infectious Diseases, ERN-RITA reference center, Copenhagen University Hospital, Rigshospitalet, Copenhagen, Denmark; 2 Institute for Immunodeficiency, Center for Chronic Immunodeficiency, Faculty of Medicine, University of Freiburg, ERN-RITA reference center, Freiburg im Breisgau, Germany; 3 Faculty of Biology, University of Freiburg, Freiburg im Breisgau, Germany; 4 Department of Clinical Immunology, Aarhus University Hospital, Aarhus, Denmark; 5 Department of Molecular Medicine, Aarhus University Hospital, Aarhus, Denmark; 6 Center for Genomic Medicine, Copenhagen University Hospital, Rigshospitalet, Copenhagen, Denmark; 7 Department of Infectious Diseases, ERN-RITA reference center, Aarhus University Hospital, Aarhus, Denmark; 8 Department of Clinical Genetics, Copenhagen University Hospital, Rigshospitalet, Copenhagen, Denmark; 9 Pediatric hematopoietic stem cell transplantation and immunodeficiency, Department of Paediatrics and Adolescents, Copenhagen University Hospital, Rigshospitalet, ERN-RITA reference center, Copenhagen, Denmark; 10 Department of Clinical Immunology, ERN-RITA reference center, Copenhagen University Hospital, Rigshospitalet, Copenhagen, Denmark; 11 Department of Clinical Medicine, University of Copenhagen, Copenhagen, Denmark

**Keywords:** Primary immunodeficiency diseases, Job syndrome, Genetic diseases, inborn, STAT3 transcription factor, Immunoglobulin E, T-Lymphocyte subsets

## Abstract

A novel heterozygous missense variant in *STAT3* (NM_139276.3: c.1250G > C, NP_644805.1: p.(Arg417Thr) was identified in a Danish family spanning five generations with diverse phenotypes consistent with autosomal dominant STAT3-Hyper-IgE-Syndrome (STAT3-HIES). Genetic analysis confirmed the absence of the variant in population databases, and computational predictions of pathogenicity were conflicting. Functional assessment revealed that the variant *STAT3* Arg417Thr maintained normal DNA binding but exhibited reduced IL-6–induced transcriptional activity and no negative-dominance. Further, significantly impaired STAT3-dependent, IL-10–mediated suppression of TNF-α production were found in patient-derived monocytes, consistent with a partial loss-of-function effect. Clinically, affected individuals presented with recurrent staphylococcal skin abscesses, eczema, atopic or infectious complications, and characteristic facial features. There was a marked variability in extra-hematopoietic manifestations such as retained primary teeth, vascular anomalies, and psychiatric disorders. Laboratory findings included elevated serum IgE levels, but only slightly reduced or normal Th17 cell counts. NIH-HIES scores varied within the family (range: 12–34), highlighting the variability of clinical expression. Based on ACMG guidelines, the variant was classified as likely pathogenic due to absence from controls, co-segregation with disease, and supportive functional evidence. This report highlights the challenges in interpreting novel *STAT3* variants and the critical need for functional validation in uncertain cases. The findings emphasize the importance of molecular diagnosis irrespective of clinical scoring and address the ethical complexities of genetic testing in familial disease.

**Supplementary Information:**

The online version contains supplementary material available at 10.1007/s10875-026-02036-8.

## Introduction

Hyper-IgE-Syndrome (HIES) is a rare inborn error of immunity (IEI), originally described as “Job’s syndrome” in the 1960s [1, 2]. It is characterized by elevated serum IgE levels, recurrent infections, and a wide range of immunological and non-immunological features [3–6]. Several genetic causes can underly a hyper-IgE-phenotype [6, 7]. Dominant negative loss-of-function variants in the signal transducer and activator of transcription 3 gene (*STAT3*) was the first identified genetic cause of autosomal dominant HIES and accounts for the majority of HIES cases [3, 4, 8, 9]. *STAT3* encodes the STAT3 protein, which plays a central role in signal transduction of a broad range of both pro- and anti-inflammatory cytokines, and is critical for IL-6 mediated differentiation of Th17 cells [5, 10]. Further, STAT3 is involved in several non-immunological pathways underpinned by the characteristic extra-hematopoietic features of STAT3-HIES including; characteristic facial features, pathological fractures, scoliosis, pneumatocele formation and vascular abnormalities [5, 11].

Contrary to loss-of-function *STAT3*-variants, gain-of-function *STAT3*-variants cause a different disease phenotype dominated by early-onset autoimmunity and lymphoproliferation [12, 13]. Adding to the complexity, *STAT3*-variants of mixed loss-of-function and gain-of-function qualities have been described [14].

Until a few years ago only a minority of the identified *STAT3*-variants in individuals with HIES had been functionally confirmed as loss-of-function [8, 15–19]. Recently, Asano et al. performed in-vitro functional analysis of predicted pathogenic *STAT3*-variants, and confirmed pathogenicity of the majority [20]. However, most of the nonsynonymous coding variants found in the general population were also predicted to be deleterious based on CADD but did not have loss-of-function effects [20]. Thus, the computational predictions of the functional impact of coding variants in *STAT3* are uncertain. This, and the phenotypic variability of affected individuals underscores the importance of functional evaluation of previously unreported *STAT3* variants.

Here, we present a Danish family spanning five generations in whom we identified a novel variant in *STAT3*, NM_139276.3: c.1250G > C, NP_644805.1: p.(Arg417Thr).) Through detailed description of the clinical presentation, genetic and immunological characterization we demonstrate that this variant exhibit partial loss-of-function qualities and underlies diverse HIES manifestations within the family.

## Methods

### Genetics

Whole genome sequencing was conducted in the two index patients from branch A and B. The method is described previously [21]. Variants were filtered using a large gene-panel related to IEI and immune function, containing > 600 genes. The identified variant was subsequently confirmed by Sanger sequencing and detected in DNA from blood from the rest of the living family members (A-III, B-III, B-IV). In two deceased family members the variant was confirmed in DNA extracted from paraffin embedded tissue (A-II), and in formalin-fixed paraffin-embedded tissue (AB-I) by next generation sequencing (TSO-500 gene-panel, Illumina [22]).

### Cell Culture

The STAT3^−/−^ cell line A4 was generated in the lab of G. Stark and provided previously to the lab of S. Ehl by T. Vogel (Department of Pediatrics, Baylor College of Medicine, Center for Human Immunobiology, Texas Children’s Hospital, Houston, TX, USA). A4 cells were cultured in McCoy’s 5a medium (Gibco, Paisley, Scotland, UK) supplemented with 5% (v/v) FCS (Anprotec, Bruckberg, Germany) and 1% (v/v) Penicillin-Streptomycin (Sigma-Aldrich, St. Louis, MO, USA) and incubated at 37 °C in humidified atmosphere containing 5% CO_2_.

### STAT3 Luciferase Reporter Assay

0.5 × 10^5^ A4 cells were seeded in 24-well plates in 0.5 ml of medium per well and transiently transfected with 250 ng of a STAT3 plasmid (or a 1:1 mixture of a STAT3 plasmid with an empty vector (EV) plasmid), 100 ng Cignal STAT3 reporter (Qiagen, Hilden, Germany) and 150 ng carrier pUC18 DNA using jetPEI transfection (Polyplus-transfection, Illkirch-Graffenstaden, France). After 42 h of incubation, cells were stimulated with 1 ng/ml or 10 ng/ml IL-6 (Peprotech, Rocky Hill, NJ, USA) or were left unstimulated for 6 h, washed with DPBS (Gibco) and harvested. Firefly and Renilla luciferase activity were measured with the Dual-Luciferase reporter assay kit (Promega, Madison, WI, USA) according to the manufacturer’s guidelines.

### DNA Binding Assay

2 × 10^6^ A4 cells were seeded in 10 cm culture dished and transiently transfected with 15 ug of a STAT3 expression plasmid using jetPEI transfection (Polyplus-transfection). After 48 h, cells were stimulated for 30 min with 100 ng/ml IL-6 (Peprotech) or left unstimulated. After stimulation, nuclear and cytoplasmatic fractions were isolated with the Nuclear Extract Kit (Active Motif, Carlsbad, CA, USA) and DNA binding activity was measured with the TransAM STAT3 DNA binding Kit (Active Motif) according to the manufacturer’s protocol.

### IL10R Assay

PBMCs were isolated from whole blood of patients (AIII, BII and BIII) and healthy controls via density gradient centrifugation using Pancoll (PAN-Biotech, Aidenbach, Germany). For each individual, five wells were seeded with 1 ml of cell suspension in a 24-well plate and incubated for 4 h at 37 °C in a humidified atmosphere containing 5% CO₂. Monocyte enrichment was achieved by gently removing non-adherent cells from the supernatant. Adherent monocytes were then stimulated overnight with recombinant human IL-10 (R&D Systems, Minneapolis, MN, USA, #217-IL/CF) at concentrations of 20 ng/ml, 5 ng/ml, or 1 ng/ml, or left unstimulated as a control. The following day, cells were stimulated with 10 ng/ml LPS (Sigma-Aldrich, L5886) for 2.5 h, harvested, and stained for surface markers using HLA-DR-PC7 (BD Biosciences, San Jose, CA, USA, #335830) and CD14-APC (BD Biosciences, #555399). For intracellular staining, cells were fixed and permeabilized using the Cytofix/Cytoperm Plus Kit (BD Biosciences, #555028) and subsequently stained for TNFα using TNFα-PE (BD Biosciences, #559321).

## Results

### Case Presentation

The family consists of two branches that were independently referred for evaluation of IEI at Copenhagen and Aarhus University Hospitals, respectively. Clinical features in both branches were consistent with HIES, prompting genetic investigation and subsequent identification of the same *STAT3* variant.

**Fig. 1:**
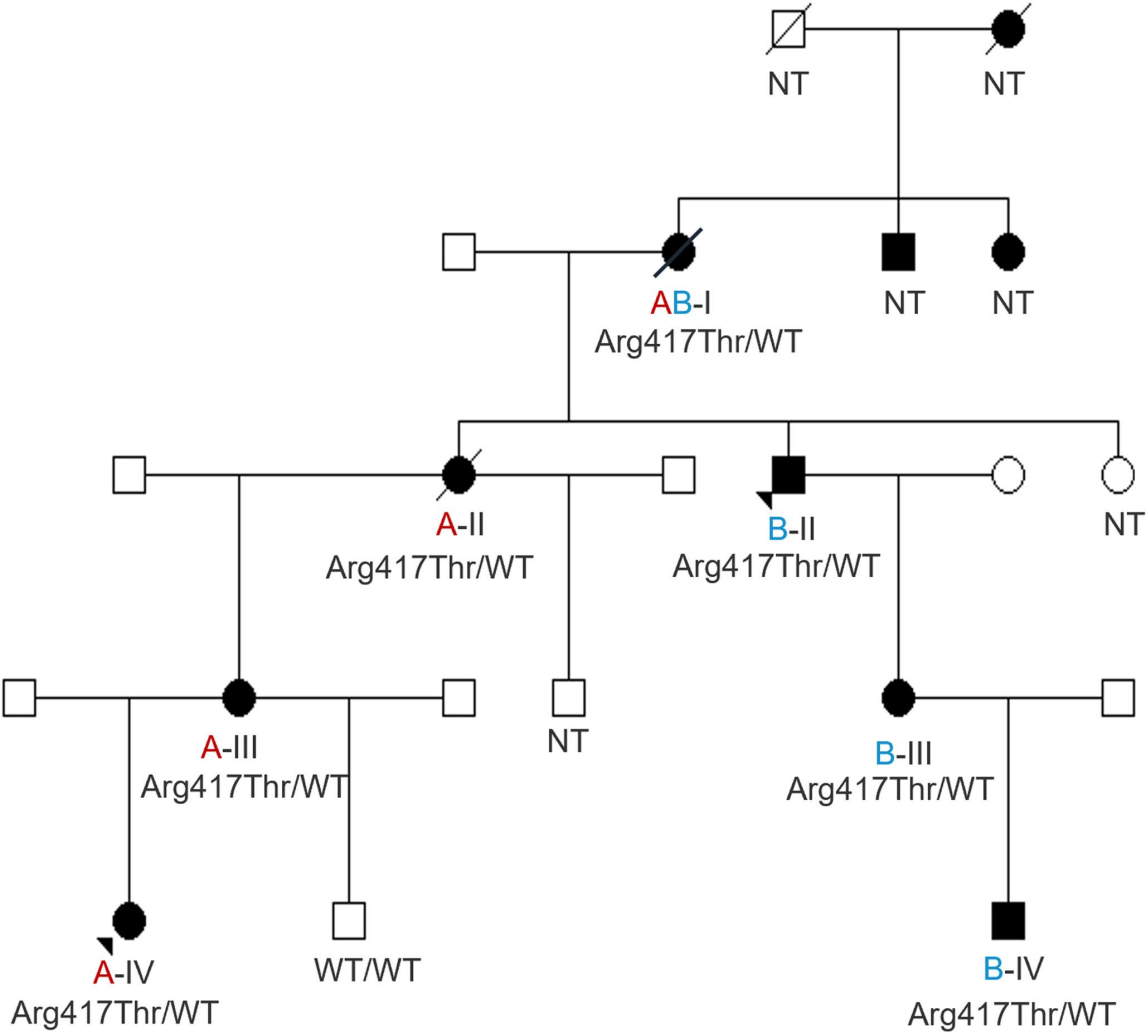
Pedigree of the family. Black color indicates individuals with Hyper-IgE-Syndrome (HIES) related symptoms. Arg417Thr/WT: Heterozygote carrier of *STAT3* NM_139276.3:c.1250G > C, NP_644805.1:p.(Arg417Thr). WT: Wild-type *STAT3.* NT: Not genetically tested. Black triangle: probands of branch A and branch B

A pedigree illustrating the affected individuals is shown in Fig. 1 and an overview of the main clinical and paraclinical findings is provided in Table 1.

### Clinical Features

#### Index Case and Maternal Family History (Branch A)

A four-year-old girl (A-IV) was referred for pediatric evaluation of possible primary immunodeficiency. She had suffered recurrent abscesses requiring antibiotics and surgical drainage, since she was two years old. *Staphylococcal aureus* was identified as the causative agent on multiple occasions. She had no history of asthma, eczema, or antibiotic-requiring upper or lower respiratory infections. Physical examination was unremarkable without identified skeletal abnormalities.

Family history revealed that her mother (A-III), grandmother (A-II), and great-grandmother (AB-I) had all suffered from recurrent skin abscesses. Further, the mother (A-III) had recurrent upper respiratory tract infections, and classical HIES facial features but no other identified skeletal abnormalities. She had previously been diagnosed with “Jobs syndrome”. For unknown reasons she had been lost to follow-up and did not attend any care for her condition.

The grandmother (A-II) died suddenly at age 34 years. She was known to have elevated plasma IgE levels and was diagnosed with HIES. An autopsy revealed severe lung disease with fibrosis, adhesions to the thoracic wall, and a pulmonary abscess (with *Streptococcus pneumonia*,* Haemophilus influenzae* and *Proteus vulgaris*). Further she had coronary atherosclerosis with stenosis. The great-grandmother (AB-I) also died young, at age 48. She had a history of malignant thymoma at the age of 25, which was treated with Adriamycin and radiation therapy. She had severe cardiomyopathy and lung fibrosis, which was perceived as a direct consequence of the thymoma treatment.

#### Second Branch of the Family (Branch B)

A 46-year-old male (B-II) was admitted with acute myocardial infarction. Coronary arteriography showed ectasia of the coronary arteries and a proximal aneurysm of the circumflex artery. His medical history included childhood atopic dermatitis, several skin abscesses, retention of primary teeth, asthma, depression, lumbar disc degeneration, sleep-apnea and irritable bowel syndrome. He had previously undergone both rheumatologic and neurologic evaluations for myalgia and elevated muscle enzymes, including muscle biopsy and genetic testing for myopathy, with normal results/findings. He mentioned that his sister (later recognized to be A-II) had “Job’s syndrome”, and his mother (AB-I), uncle, aunt, and grandmother all had recurrent infections. IgE was subsequently found markedly elevated. Classical HIES facial features were present. Imaging showed no scoliosis, pneumatoceles or cerebral aneurysms.

His adult daughter (B-III) had a lifelong history of eczema and impetigo, recurrent otitis media and externa (with growth of *S. aureus* and *Candida albicans* on multiple occasions), rosacea, bilateral mastitis postpartum, and retention of primary teeth. A CT coronary angiogram showed normally calibrated coronary arteries. The newborn son of B-III (B-IV) had impetigo with *S. aureus*.

**Table 1:** Clinical and paraclinical findings of the family

	A-IV	A-III	A-II	AB-I	B-II	B-III	B-IV
Clinical features							
Age at evaluation (years)	**6**	**30**	**36**	**48**	**46**	**25**	**0**
Skin abscesses	Yes	Yes	Yes	Yes	Yes	No	No
Other Infections		Upper respiratory tract infections	Pneumonia (lung abscess), Recurrent Zoster infection	Oral and vaginal candidiasis	Upper respiratory tract infections, pneumonia	Pneumonia, internal and external otitis, mastitis, oral candidiasis	Impetigo
Characteristic face	Yes	Yes	Yes		Yes	Yes	NA
Skeletal abnormalities	No	No	Osteoporosis		Lumbar disc degeneration, myalgia	No	No
Skin			Eczema		Newborn rash, eczema	Newborn rash, eczema, rosacea	Newborn rash, eczema
Retention of primary teeth	No (delayed development)	No			Yes	Yes	
Others	ADHD	Anxiety	Hidradenitis, Asthma Depression	Malignant thymoma, cardiomyopathy and lung fibrosis	Depression, asthma, irritable bowel syndrome, sleep-apnea, myocardial infarct, myalgia		
Paraclinical findings							
Highest serum-IgE level (IU/mL) (< 150)	**700** (< 60)	**13**,**300**	**80**,**000**		**3**,**368**	**3**,**816**	**20** (< 15)
IgA (g/L) (0.7–4.3)	0.38 (0.3–2.22)	1.18			1.27	1.9	
IgG (g/L) (6.1–14.9)	8.3 (5.0-11.7)	9.4			11.1	11.6	
IgM (g/L) (0.39–2.08)	0.95 (0.56–2.31)	0.92			1.12	1.31	
T-cell proliferation	Normal				Normal		
DHR	Normal						
STAT3 phosphorylation	Normal	Normal			Normal	Normal	Slightly reduced
Lymphocytes	lymphocytosis (5.5 10^9/L)	Normal			Normal	Normal	
T-cells (10^9/L) (0.69–2.70)	**3.7** (1.20–2.60)	1.6			0.78	1.6	
CD4-T-cells (10^9/L) (0.39–1.70)	**2.2** (0.53–1.30)	0.98			43% of T	61% of T	
CD8-T-cells (10^9/L) (0.19–1.03)	**1.2** (0.33–0.92)	0.59			48% of T	29% of T	
B-cells (10^9/L) (0.09–0.57)	**1.5** (0.27–0.86)	0.38			0.14	0.12	
NK-cells (10^9/L) (0.08–0.56)	0.22 (0.07–0.48)	0.13			0.24	0.09	
Th17-cells	**0.2**% of CD3posCD4pos-cells (normal range not established, lower than healthy controls)	**0**,**53**% of CD4 + CD45RO+ cells (normal range (0,6 − 3,2%))			0.57% of CD3posCD8neg-cells (normal range (0.21–1.7%))	0.66% of CD3posCD8neg-cells (normal range (0.21–1.7%))	
Lung function	Normal	Normal			Normal		
Lung parenchyma		No abnormalities	Lung fibrosis, adhesions, lung abscess	Lung fibrosis	No Abnormalities	Normal, except few para-septal bullae	
Vascular abnormalities			Atherosclerosis (coronary and periphery)		Coronary aneurism, No cerebral aneurism	No coronary aneurisms	
Total NIH HIES score	**14**	**24**	**25**	NA	**34**	**25**	**12**
Simplified HIES score	2	2	2	NA	15	8	4

*STAT3 NM_139276.3:c.1250G>C, NP_644805.1:p.(Arg417Thr). If the information is unknown the field is blank. The reference values indicated in the first column are for adults. Age specific references are represented in the columns for the children (A-IV and B-IV), where applicable. Abnormal results are highlighted in bold. NIH HIES score: National Institutes of Health (NIH) Hyper IgE Syndrome (HIES) scores [4]. A score of ≥ 20 is usually used as the cut-off for suggestive HIES and ≥ 40 for probable HIES. The simplified HIES score, is based on 5 cardinal clinical features: recurrent pneumonia, newborn rash, pathologic bone fractures, characteristic face and high palate [23]. Here a score of > 30 together with IgE levels of > 1000 is indicative of HIES

### Paraclinical Findings and National Institute of Health (NIH) HIES score

All tested family members had elevated IgE levels for their age. Eosinophilia was not found. Lymphocyte counts and distribution was generally normal, with normal levels of immunoglobulins. Th17 cells were conducted with three different assays, where normal ranges are established for two of the patients. B-II and B-III had normal Th17 cell fractions, whereas A-III was just below the normal range. A-IV had a lower fraction compared to two (adult) healthy controls. In vitro stimulation of PBMCs with human IL-6 at various concentrations, followed by intracellular STAT3 phosphorylation analysis via flow cytometry, revealed slightly reduced phosphorylation for the B-IV, but no significant differences between the other tested patients (A-III, A-IV, B-II, B-III) and healthy controls (Table 1 and Supplement Fig. 1).

According to the NIH HIES scoring system [4] the girl (A-IV) scored 14 points while the mother (A-III) and grand-mother (A-II) scored 24 and 25 points respectively. Branch B had NIH-HIES scores of 34, 25 and 12, respectively (Table 1 and Supplement Table 1).

### Genetics

**Fig. 2:**
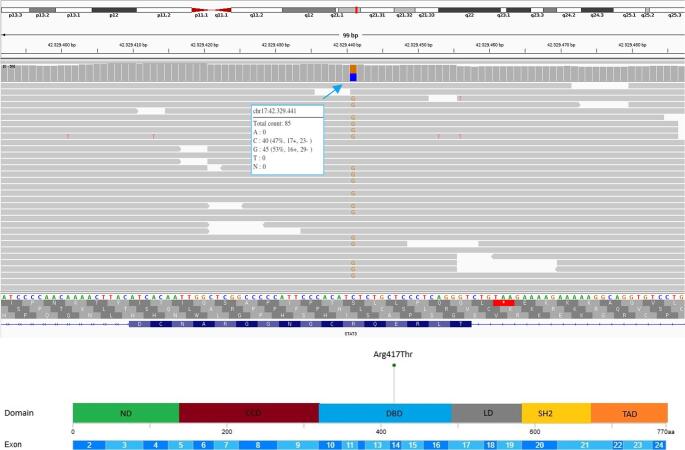
Whole genome sequencing of the *STAT3* NM_139276.3:c.1250G > C, NP_644805.1:p.(Arg417Thr). The variant is in the DNA-binding-domain (DBD). ND: N-terminal domain, CCD: coiled-coil domain, LD: linker domain, SH2: Src homology 2 domain, TAD: Transactivation domain. Domain figure adapted from https://www.cbioportal.org/mutation_mapper [25]

A heterozygote missense variant in *STAT3*, NM_139276.3: c.1250G > C, NP_644805.1:p.(Arg417Thr) was identified (Fig. 2). The variant is localized in the DNA-binding domain. The variant is absent from population database (gnomAD) [24]. The variant changes a moderately conserved amino acid. In silico predictions are conflicting (Align GVGD: Class Co, SIFT: Tolerated, MutationTaster: Disease causing, PolyPhen-2: Possible damaging, REVEL: Deleterious) and the Combined Annotation Dependent Depletion (CADD) score is 27.9.

The variant was identified in the living family members A-IV, A-III, B-II, B-III and B-IV, and post-mortem in A-II and AB-I. A newborn half-brother to A-IV did not carry the variant. The family had no contact to two reportedly healthy family members (the half-brother of A-III, and the sister of A-II and B-II) why these individuals were unavailable for genetic testing (Fig. 1).

### STAT3 Transcriptional Activity and DNA Binding

**Fig. 3:**
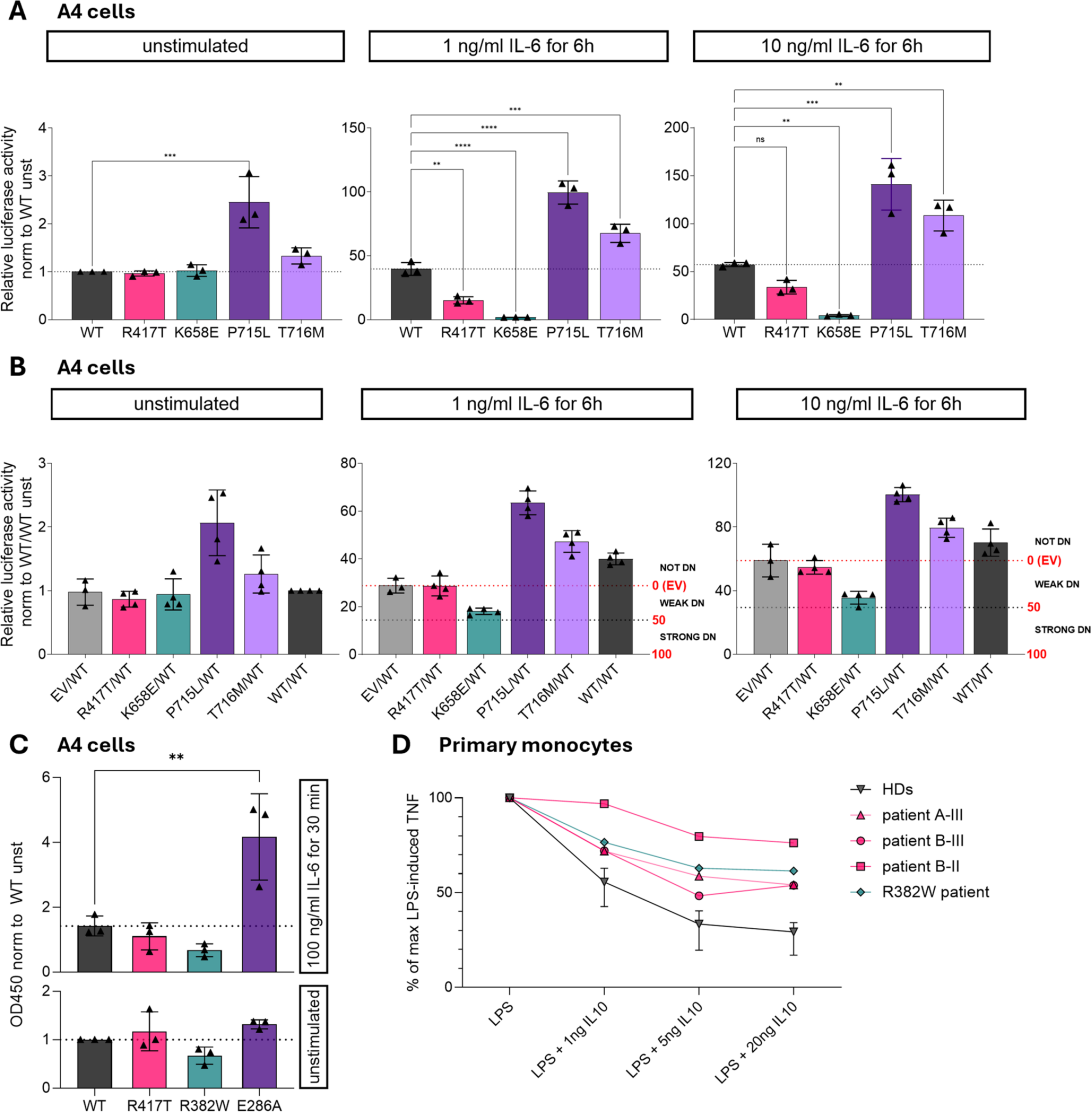
Functional consequences of the STAT3 Arg417Thr (R417T) variant. **A** STAT3 specific transcriptional activity assessed by a luciferase reporter assay in A4 cells reconstituted with different STAT3 alleles. Graphs show the relative luciferase activity (mean ± SD of 3 individual experiments) compared to WT without IL-6 stimulation. Previously reported STAT3 loss-of-function (LOF) Lys658Glu (K658E) and STAT3 GOF Pro715Leu (P715L) and Thr716Met (T716M) variants were analyzed as controls. Statistical analysis by Ordinary one-way ANOVA with Dunnett’s multiple comparisons test. *p < 0.05; **p < 0.01; ***p < 0.001; ****p < 0.0001. **B** STAT3 specific transcriptional activity assessed by a luciferase reporter assay in A4 cells reconstituted with a 1:1 mixture of the STAT3 wildtype allele with an empty vector (EV) or different STAT3 alleles. Graphs show the relative luciferase activity (mean ± SD of 3 individual experiments) compared to WT without IL-6 stimulation. Transcriptional activity of IL-6-stimulated EV/WT cells is indicated by a red dashed line. 50% of EV/WT activity is indicated by a black dashed line. Variants with activity below the EV/WT activity are considered to be DN, while variants above the EV/WT activity are considered to be not DN. **C** DNA binding activity to a STAT3-responsive probe (3’-TTCCCGGAA-5’) in a DNA binding ELISA performed with nuclear cell extracts from reconstituted A4 cells. Graphs show the OD450 (mean ± SD of 3 individual experiments) normalized to WT without IL-6 stimulation. Previously reported STAT3 LOF Arg382Trp (R382W) and STAT3 GOF Glu286Ala (E286A) variants were analyzed as controls. Statistical analysis by Ordinary one-way ANOVA with Dunnett’s multiple comparisons test. *p < 0.05; **p < 0.01; ***p < 0.001; ****p < 0.0001. **D** STAT3 mediated reduction of TNF production as determined by flow cytometry of primary monocytes of patients A-III, B-II, B-III, 6 HD controls and a STAT3 HIES patient Arg382Trp (R382W). Graph shows median with range of the percentage of maximal LPS-induced TNF production upon LPS + 0ng/ml, 1, 5 or 20 ng/ml IL-10 for HDs and individual values for all patients

To investigate the functional impact of the *STAT3* Arg417Thr variant, we conducted a luciferase reporter assay in STAT3-deficient A4 cells. Cells were transfected with either *STAT3* wild-type (WT), the Arg417Thr variant, the known Pro715Leu or Thr716Met gain-of-function (GOF) or the HIES-associated Lys658Glu loss-of-function (LOF) *STAT3* variants, along with a STAT3-responsive luciferase reporter. Following interleukin-6 (IL-6) stimulation or no treatment, STAT3 transcriptional activity was assessed by quantification of luciferase activity (Fig. 3A). Under basal (unstimulated) conditions, the Arg417Thr variant exhibited transcriptional activity comparable to *STAT3* WT. However, upon low-dose IL-6 stimulation (1 ng/ml), the variant *STAT3* Arg417Thr caused significantly reduced transcriptional activity compared to WT. A similar trend was observed at higher IL-6 concentrations (10 ng/ml), although the difference was less pronounced. As expected, the GOF variants Pro715Leu and Thr716Met showed enhanced activity, while the LOF variant Lys658Glu displayed markedly reduced activity, confirming the assay’s validity.

To assess whether the *STAT3* Arg417Thr variant exerts a dominant-negative effect, we performed a co-transfection assay in STAT3-deficient A4 cells. Cells were transfected with a 1:1 mixture of *STAT3* WT together with either empty vector (EV), Arg417Thr, the LOF Lys658Glu variant, or the GOF variants Pro715Leu or Thr716Met, alongside a STAT3-responsive luciferase reporter (Fig. 3B). STAT3-dependent transcriptional activity was quantified under basal conditions and following interleukin-6 (IL-6) stimulation (1 or 10 ng/ml). EV/WT served as a baseline for the effect of a single transfected allele; all other conditions reflect the influence of a second allele on WT activity. Co-expression of Arg417Thr with WT (R417T/WT) yielded luciferase activity indistinguishable from EV/WT across all conditions, indicating no detectable dominant-negative effect of Arg417Thr on WT STAT3. As expected, K658E/WT showed reduced activity consistent with a dominant-negative effect, whereas both GOF/WT conditions (P715L/WT and T716M/WT) exhibited increased activity upon IL-6 stimulation, confirming the assay’s dynamic range and internal controls.

To ensure that the variant is expressed in A4 cells, we performed a Western Blot analysis, which confirmed both its basal expression and normal phosphorylation upon IL-6 stimulation (Supplement Fig. 2).

As the Arg417Thr variant is located within the DNA-binding domain of *STAT3*, we evaluated its impact on DNA binding capacity. STAT3-deficient A4 cells were transfected with *STAT3* WT, Arg417Thr, or control variants and either stimulated with 100 ng/ml IL-6 or left unstimulated. Nuclear and cytoplasmic fractions were isolated, and DNA binding activity in the nuclear fraction was assessed using an immobilized DNA probe (3′-TTCCCGGAA-5′) (Fig. 3C). The Arg417Thr variant exhibited normal DNA binding under basal conditions and slightly reduced (non-significant) binding following IL-6 stimulation. In contrast, the known LOF variant Arg382Trp showed no increase in DNA binding upon stimulation, while the GOF variant Glu286Ala displayed significantly elevated binding after stimulation.

### IL-10-Mediated Reduction of TNF Production Via STAT3 in monocytes

PBMCs from patients were incubated overnight with increasing concentrations of interleukin-10 (0, 1, 5, or 20 ng/ml) followed by stimulation with LPS to induce TNF-α production. In healthy monocytes, IL-10 activates the STAT3 signalling pathway, resulting in a dose-dependent suppression of LPS-induced TNF-α release [26]. In this assay, the extent of TNF-α reduction serves as a functional readout of STAT3 activity. All patient samples exhibited impaired IL-10–mediated suppression of TNF-α production across all tested concentrations to a similar extent as the known *STAT3* LOF variant Arg382Trp (Fig. 3D).

## Discussion

In a family of five generations of individuals with diverse presentation of HIES symptoms, we identified a novel variant in *STAT3* Arg417Thr in four generations. Functional assessment revealed that *STAT3* Arg417Thr maintained normal DNA binding but exhibited reduced IL-6–induced transcriptional activity and significantly impaired STAT3-dependent, IL-10–mediated suppression of TNF-α production in patient-derived monocytes, consistent with a partial loss-of-function effect. No dominant negative effect of the variant could be demonstrated, which supports haploinsufficiency as the underlying mechanism.

Upon the confirming functional analysis and the extended co-segregation information we classify the c.1250G > C, p.Arg417Thr variant as likely pathogenic based on the following ACMG-criteria: Absent in population databases (PM2_supporting), computational evidence (REVEL) support a deleterious effect (PP3_supporting), missense change at an amino acid residue, where different pathogenic missense changes have previously been described (PM5_supporting), co-segregation with disease (PP1_strong), missense variant in a gene that has a low rate of benign missense variations (PP2) and established functional studies show a deleterious effect (PS3_supporting). According to ACMG guidelines, these criteria yield 9 points, which corresponds to a likely pathogenic variant (class 4). 10 points are required to classify the variant as pathogenic (class 5).

Variants of uncertain significance are a frequent clinical challenge, reported in over 70% of all clinically reported missense variants in ClinVar [27, 28]. As underscored in the study by Asano et al. 2021 [20] there is a need of functional verification of novel variants in *STAT3*. However, it requires significant work to functionally assess pathogenicity, as well as expensive laboratory equipment. Thus, a collaborative effort is warranted to overcome this hurdle.

Variant classification is of clinical importance. Besides verification of the molecular diagnosis and specific disease management, it is essential for reproductive genetic counselling and decisions regarding invasive prenatal testing or preimplantation genetic testing. In this regard, likely pathogenic and pathogenic variants are both considered as confirmatory genetics, whereas variants of uncertain significance make counselling extremely challenging, and prenatal or preimplantation genetic testing is not an option.

The two branches of the family live in separate locations and do not have regular contact. The familiar connection between the branches only became apparent as the variant from branch A was registered in ClinVar [28]. Thus, contact between the two diagnosing centers could be established upon identification of the same variant in branch B. This underscores the importance of sharing variant information, but also highlight ethical challenges as it was a delicate situation to unravel the family history.

The family presented with classical manifestations of STAT3-HIES such as elevated IgE-levels and distinctive facial features. The dominating infectious symptom was recurrent skin abscesses with *S. Aureus*. Respiratory infections were frequently reported in the family, though dominated by upper respiratory tract infections. Other than these common features, there was great within-family phenotypic variability, especially of the non-hematopoietic features, such as retained primary teeth, coronary artery aneurisms and eczema/newborn rash, although, the latter could be subjected to recall bias. Skeletal and connective tissue abnormalities, such as hyperextensibility, scoliosis and pathological fractures were absent in the family.

The variable expressivity of symptoms is reflected in the NIH-score which were generally low and ranged from 12 to 34. A score of ≥ 20 is usually used as the cut-off for suggestive HIES and ≥ 40 for probable HIES. Although the probable cut-off of ≥ 40 is met in many HIES-patients [23], not all HIES patients have a high NIH-HIES score. In some HIES cohorts, only a minority of patients have a score ≥ 40 [29, 30]. A simplified score of five cardinal symptoms: pneumonia; pathological fractures, characteristic face, newborn rash and high plate with a score of ≥ 30 has also been used [23]. The presented family of this study did not meet the cut-off for probable HIES in either scheme. However, there is a great diversity in clinical presentations between HIES-cohorts and even with-in families [29–32], emphasizing the variable expressivity of this disease. Therefore, a genetic diagnosis should be pursued in case of suspicion of HIES regardless of clinical HIES-score. We cannot firmly conclude on the penetrance of this Arg417Thr variant as only one healthy family member was available for genetic testing. However, our findings suggest high penetrance in line with other *STAT3* variants associated with AD-HIES [16].

Four of the family members were diagnosed with neuropsychiatric disorders (Table 1). The literature on psychiatric disorders among HIES patients is scarce, but depression was frequently reported in the HIES cohort of the USIDNET registry (18/82 (22%)) [32]. On the other hand, psychiatric disorders are frequent in the general population [33] and often runs in families [34], so whether this is related to HIES or is coincidental remains unknown.

Our functional analyses focused on the IL-6– and IL-10–STAT3 axes, which are directly relevant to the immunological manifestations of STAT3-HIES. Importantly, experiments performed in primary monocytes from affected individuals demonstrated a clear loss-of-function effect in the IL-10–STAT3 axis, evidenced by clearly impaired STAT3-dependent suppression of TNF-α production. This provides physiologically relevant evidence that the Arg417Thr variant compromises STAT3 function at endogenous expression levels.

In contrast, other functional assays relied on overexpression-based cell line models and were designed to interrogate defined signaling steps rather than to model STAT3 function in vivo. These approaches do not recapitulate the heterozygous state, tissue-specific regulation, or endogenous feedback mechanisms. Furthermore, stimulation with selected cytokines (IL-6 and IL-10) at supraphysiological concentrations may underestimate functional impairment under physiological conditions. Moreover, luciferase reporter and DNA-binding assays assess STAT3 activity at single consensus motifs and may not fully capture gene- or chromatin-context–dependent transcriptional effects. Notably, the DNA-binding assay was primarily optimized to detect gain-of-function variants and may be less sensitive to partial loss-of-function effects.

Finally, our analyses did not address other STAT3-dependent pathways that may be particularly relevant for non-immune manifestations. The pronounced intra-familial phenotypic variability suggests that STAT3 genotype alone does not fully determine disease expression. It is likely that environmental factors, modifier genes, epigenetic variation, amount of STAT3 protein, or differential activation of STAT3 pathways downstream of different cytokines contribute to clinical heterogeneity. It has also been shown that in cases of suspected STAT3 haploinsufficiency, extra-hematological symptoms were less pronounced or even absent, associated with a milder, more atypical and predominantly infection-driven phenotype [35–38]. In this context, strong dominant-negative STAT3 variants may drive more penetrant extra-hematopoietic features, whereas partial loss-of-function variants - potentially reflecting STAT3 haploinsufficiency -, such as Arg417Thr, may permit additional modifiers to play a more prominent role in shaping the clinical phenotype.

In conclusion, we demonstrate that the previously unreported *STAT3* variant, c.1250G > C, p.Arg417Thr, confers partial loss-of-function qualities and causes HIES in a large Danish family with variable expressivity. Confirming the molecular diagnosis ensured correct clinical care and genetic counseling for the family. The study highlights the importance of pursuing and validating a molecular diagnosis as well as sharing variant details. Further it stresses the clinical and ethical complexities inherent to genetic testing.

## Supplementary Information

Below is the link to the electronic supplementary material.

Supplementary Material 1

## Data Availability

No datasets were generated or analysed during the current study.
