# Supplementary material for: Novel Partial Loss-of-function STAT3-variant as Cause of Hyper-IgE-Syndrome in a Danish Family with Variable Expressivity
Source: J Clin Immunol. 2026 Jun 8;46(1):80. doi: 10.1007/s10875-026-02036-8 (PMC13461928; doi:10.1007/s10875-026-02036-8)
Supplement: Supplementary file 1 [file 10875_2026_2036_MOESM1_ESM.docx]

**Supplement material** Novel partial loss-of-function *STAT3*-variant as cause of Hyper-IgE-Syndrome in a Danish family with variable expressivity.

| Supplement Table 1 | A-IV | A-III | A-II | B-II | B-III | B-IV |
| --- | --- | --- | --- | --- | --- | --- |
| Age at evaluation | 6 | 30 | 36 | 46 | 25 | 0 |
| Highest serum-IgE level (international units/mL) | 700 (= 4 point) | 13.3000 (= 10 point) | 80.000 (= 10 point) | 3.368 (= 10 point) | 3.816 (=10 point) | 20 (=0 point) |
| Skin abscesses | >4 (= 8 point) | >4 (= 8 point) | (>4 = 8 point) | 1-2 (=2 point) | 0 | 0 |
| Pneumonia (episodes over lifetime) | 0 | 0 | 1 (= 2 point) | 3 (=6 point) | 1(=2 point) | 0 |
| Parenchymal lung anomalies | 0 | 0 | 0 | 0 | 0 |  |
| Retained primary teeth | Delayed teeth development | 0 |  | 3 (= 4 point) | 3 (= 4 point) |  |
| Scoliosis, maximum curvature | 0 | 0 |  | 0 | 0 | 0 |
| Fractures with minor trauma | 0 | 0 |  | 0 | 0 | 0 |
| Highest eosinophil count (cells/microL) | 0 | 0 |  | 0 | 0 | 0 |
| Characteristic face | Mildly present (=2 point) | Mildly present (=2 point) |  | Present (=5 point) | Mildly present (= 2 point) | 0 |
| Midline anomaly | 0 | 0 |  | 0 | 0 | 0 |
| Newborn rash | 0 | 0 |  | Present (=4 point) | Present (=4 point) | Present (=4 point) |
| Eczema (worst stage) | 0 | 0 | Mild (=1 point) | Moderate (=2 point) | Moderate (=2 point) | Mild (=1 point) |
| Upper respiratory infections per year | 0 | >6 (= 4 point) |  | 0 | 0 | 0 |
| Candidiasis | 0 | 0 |  | 0 | Oral (=1 point) | 0 |
| Other serious infections | 0 | 0 | Severe (=4 point) | 0 | 0 | 0 |
| Fatal infection | 0 | 0 |  | 0 | 0 | 0 |
| Hyperextensibility | 0 | 0 |  | 0 | 0 | 0 |
| Lymphoma | 0 | 0 |  |  | 0 | 0 |
| Increased nasal width |  |  |  | 1-2 SD (= 1 point) | 0 |  |
| High palate | 0 | 0 |  | 0 | 0 | 0 |
| Young-age correction | 0 | 0 | 0 | 0 | 0 | < 1 year (= 7 point) |
| Total NIH HIES score | 14 | 24 | 25 | 34 | 25 | 12 |
| Simplified HIES score | 2 | 2 | 2 | 15 | 8 | 4 |

**Supplement table 1** National Institutes of Health (NIH) Hyper IgE Syndrome (HIES) scores of the family [1], A score of ≥20 is usually used as the cut-off for suggestive HIES and ≥40 for probable HIES. The simplified HIES score, is based on 5 cardinal clinical features: recurrent pneumonia, newborn rash, pathologic bone fractures, characteristic face and high palate [2]. Here a score of >30 together with a IgE level of > 1000 is indicative of HIES. If the information is unknown the field is blank

1. Grimbacher B, Schäffer AA, Holland SM, Davis J, Gallin JI, Malech HL, et al. Genetic linkage of hyper-IgE syndrome to chromosome 4. Am J Hum Genet. 1999;65:735–44.

2. Woellner C, Gertz EM, Schäffer AA, Lagos M, Perro M, Glocker E-O, et al. Mutations in STAT3 and diagnostic guidelines for hyper-IgE syndrome. J Allergy Clin Immunol. 2010;125:424-432.e8. https://doi.org/10.1016/j.jaci.2009.10.059

**Supplement Figure 1: STAT3 phosphorylation**. Flow cytometry results of STAT3 phosphorylation of peripheral blood mononuclear cells of patient B-II, B-III, B-VI, A-III, A-IV and healthy controls. STAT3-phosphorylation was measured unstimulated and in-vitro IL-6 stimulated at different IL6-consentrations. No significant differences between the patients and controls were observed, although B-IV had slightly reduced STAT3 phosphorylation compared to the corresponding controls.

**Method: Intracellular STAT3 Phosphorylation Assay** Peripheral blood mononuclear cells (PBMCs) were thawed and resuspended at 1 × 10^6^/ml in sterile PBS. Aliquots of 400 µl were stimulated for 15 min at 37 °C with recombinant human IL-6 (Peprotech) at final concentrations of 10, 1, 0.1, or 0.01 ng/ml (B-II, BIII and B IV) or 10 and 100 ng/ml (A-III and A-IV), or left unstimulated. Cells were fixed with 500 µl Fix Buffer (BD, cat. no. 554655) for 10 min at 37 °C, washed twice with 0.1% BSA/PBS, and surface-stained with anti-CD4 [PE-Cy7] (BD, cat. no. 557852) and anti-CD3 [BV421] (BioLegend, cat. no. 300434) for 15 min at room temperature in the dark. Following permeabilization with Perm Buffer IV (BD) for 20 min at room temperature, cells were washed twice and stained intracellularly with anti-STAT3 (pY705) [Alexa Fluor 488] (BD, cat. no. 557814) for 30 min at room temperature in the dark. STAT3 phosphorylation was analyzed in gated CD3+CD4+ T cells.

tSTAT3

Tubulin

WT

R417T

K658E

-

+

-

+

-

+

IL-6 (10 ng/ml) for 30 min

pSTAT3

**Supplement Figure 2: STAT3 Arg417Thr expression and phosphorylation in reconstituted A4 cells**. Immunoblotting of phosphorylated STAT3-Y705 (pSTAT3) and total STAT3 (tSTAT3) in A4 cells reconstituted with STAT3 WT, STAT3 Arg417Thr and STAT3 Lys658Glu alleles. No differences in STAT3 expression and phosphorylation were observed.

**Method: Phospho-STAT3 Immunoblotting.** 1 x 10^5^ A4 cells were seeded in 24-well plates in 1 ml of medium per well and transiently transfected with 500 ng of a STAT3 expression plasmid using jetPEI transfection (Polyplus-transfection). After 48 hours, cells were stimulated for 30 minutes with 10 ng/ml IL-6 (Peprotech) or left unstimulated. Cells were then lysed in IPN150 lysis buffer (50 mM TRIS/HCl pH 8, 150 mM NaCl, 5 mM MgCl2, 0.1% NP-40, 1 mM DTT, 1X c0mplete protease inhibitor (Roche), 1% Phosphatase Inhibitor Cocktail 2 and 3 (both Sigma)) and separated by size on a 10% acrylamide gel before transfer to nitrocellulose membranes. Membranes were blocked for 15 min at room temperature (RT) in EveryBlot Blocking Buffer (Bio-Rad) and incubated with the following primary antibodies in 3-5 % BSA for 1 h at RT or overnight at 4°C: pSTAT3 (#9145S, Cell Signaling), tSTAT3 (#9139S, Cell Signaling) and α-Tubulin (#3973, Cell Signaling). Membranes were then incubated with anti-mouse or anti-rabbit HRP-conjugated secondary antibodies (both JacksonImmunoResearch) in 5% nonfat dry milk for 1 h at RT. Bands were visualized with ECL Select Western Blotting Detection Reagent (Cytiva).
